# Mammary analogue secretory carcinoma of the salivary glands

**DOI:** 10.3332/ecancer.2023.1511

**Published:** 2023-02-23

**Authors:** Lucas Avondet, Roque Adan, Barbara M Berenstein, Ismael Tayagui, Gonzalo Zeballos, Erica Rojas Bilbao, Fernanda Alsina, Nicolas Bustos

**Affiliations:** 1 Department of Surgery, Head and Neck Unit, Angel H Roffo Oncology Institute (IOAHR), C1417 CABA, Buenos Aires, Argentina; 2 Department of Anatomical Pathology, Angel H Roffo Oncology Institute (IOAHR), C1417 CABA, Buenos Aires, Argentina; 3 Department of Diagnostic Imaging, Angel H Roffo Oncology Institute (IOAHR), C1417 CABA, Buenos Aires, Argentina

**Keywords:** salivary glands, mammary secretory carcinoma, molecular pathology, ETV6-NTRK3 translocation

## Abstract

**Background:**

Mammary analogue secretory carcinoma (MASC) is a new disease among tumours affecting the salivary glands. It was first reported in 2010, and few cases have been reported worldwide. MASC is often incorrectly diagnosed as salivary gland acinic cell carcinoma. We present here the case of a patient with an asymptomatic parotid tumour who underwent a parotidectomy of the superficial lobe.

**Case report:**

A 78-year-old female patient came to the clinic for a tumour of approximately 2.5 × 2.5 cm and a hard, elastic consistency that had grown insidiously in the right preauricular region. Magnetic resonance imaging of the head and neck showed a heterogeneous ovoid lesion located in the lower part of the superficial lobe of the right parotid gland, measuring 29 × 27 × 27 mm. A superficial parotidectomy was performed with the facial nerve identified and preserved. Immunohistochemistry was positive for S100, mammaglobin, periodic acid Schiff (PAS) and GATA-3. Fluorescence in situ hybridisation analysis was subsequently performed and Translocation-ETS-Leukemia Virus (ETV6) gene rearrangement observed. These findings were consistent with diagnosis of a MASC. The patient then required no new interventions or adjuvant therapy. At publication, she was free of disease and continues in clinical follow-up.

**Conclusion:**

MASC is a tumour of the saliva glands that is recently described and rare. There are no studies that describe its biological behaviour or prognosis precisely.

## Introduction

The salivary glands are affected by a wide range of neoplasms [1]. This variety complicates accurate morphological characterisation and timely histological diagnosis [2]. In 2010, Skálova *et al* [3] first described mammary analogue secretory carcinoma (MASC), a new disease in the differential diagnosis of salivary glands neoplasms. MASC shares morphological and immunohistochemical characteristics with mammary secretory carcinoma. It affects both genders, but is slightly more common in males. Age at onset is about 40 years, but with a wide range (15–70 years) [2–3]. Most MASC cases affect the parotid gland, but there are reports of lesions in the submandibular and minor salivary glands, mainly in the oral cavity. MASC most frequently presents as a painless nodule that grows slowly but progressively [4–5].

It is associated with a chromosome translocation, t(12;15) (p13;q25), that results in Translocation-ETS-Leukemia Virus (ETV6) - Neurotrophic Tyrosine Receptor Kinase (NTRK3) gene fusion [2, 3, 6]. This mutation is not observed in other salivary gland tumours and its identification makes MASC diagnosis possible [3].

## Case report

**Figure 1.**
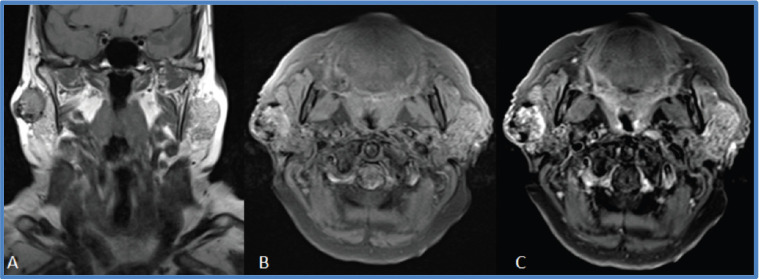
Coronal MRI section in T1 sequence. (a). Right superficial parotid nodule with a heterogeneous signal, well-defined edges and an apparently fibrous capsule. (b and c): Axial MRI section in T1 FAT SAT sequence with and without gadolinium. This nodule shows a predominantly peripheral enhancement after gadolinium administration.

**Figure 2.**
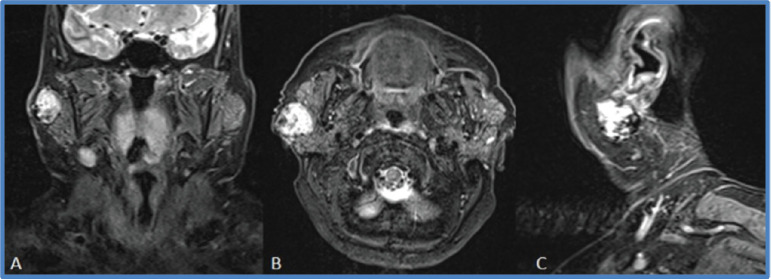
MRI sections. (a): coronal, (b): axial and (c) sagittal in STIR sequence. Right superficial parotid nodule, with well-defined edges, a predominantly hyperintense, heterogeneous signal and papillary-like projections inside.

A 78-year-old woman presented with a painless tumour in the right parotid region that had grown for 8 years, but with no associated signs and symptoms. She had a 50-year history of smoking (10 pack-years) and did not report alcohol consumption. Physical examination found a mobile, elastic, hard, ovoid lesion in the right preauricular region. Magnetic resonance imaging (MRI) showed a right superficial parotid nodule with well-defined edges and a fibrous capsule that was hypointense in nearly all sequences, but with a mainly hyperintense, heterogeneous signal in T2 sequences. This nodule showed enhancement with intravenous contrast and papillary-like projections inside. It measured 29 × 27 × 27 mm and was first interpreted as a primary neoformative process (Figures 1 and 2).

**Figure 3.**
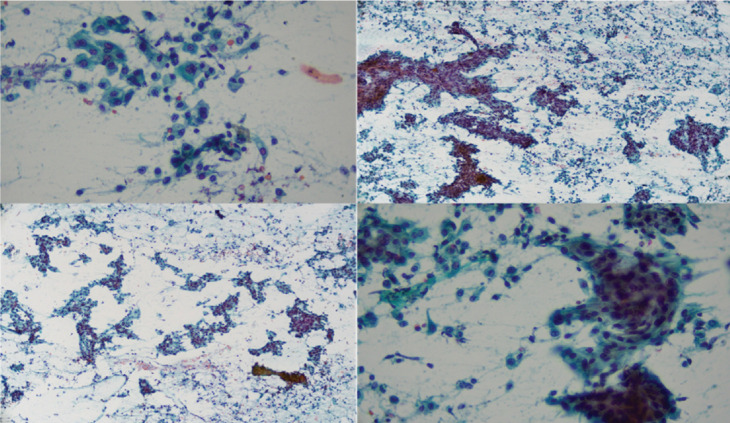
FNA cytology: Bunches of atypical epithelial cells arranged in layers and papillary structures with notable macrokaryosis and scarce cytoplasm.

Fine needle aspiration (FNA) was performed on the lesion. Neoplastic cells were observed with moderate atypia, macrokaryosis, occasional nucleoli and scant cytoplasm arranged in three-dimensional strips, with nuclear superimposition in a haematic background in which few lymphocytes were identified (Milan Classification V) (Figure 3).

**Figure 4.**
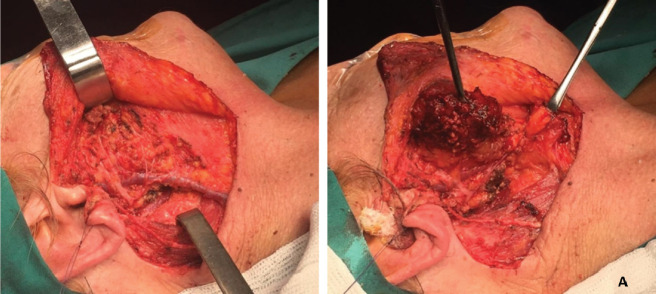
(a): Surgical procedure. Right superficial parotidectomy. Image shows a tumour lesion that affects nearly all the superficial lobe of the parotid gland. (b): Surgical site. This image shows anatomical integrity of the facial and great auricular nerves and the external jugular vein. This result features an ideal cleavage plane and separation from the tumour lesion.

**Figure 5.**
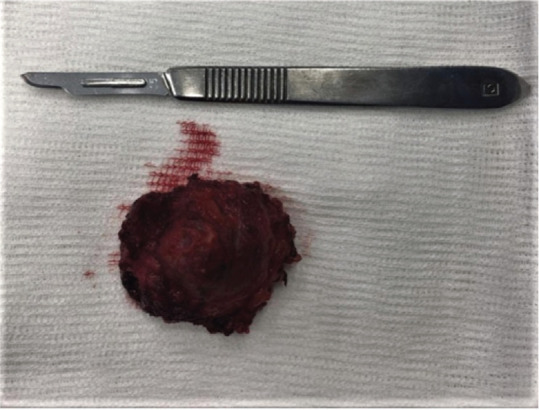
Surgical specimen. Encapsulated lesion, hard, elastic to touch, approximately 25 × 22 mm.

A right superficial parotidectomy was performed, with the facial nerve identified and preserved (Figure 4a and b). Intraoperative analysis of the surgical specimen identified low-grade monomorphic epithelial proliferation with margins free of tumour lesion (Figure 5). The patient tolerated the procedure well and left the hospital after 24 hours.

**Figure 6.**
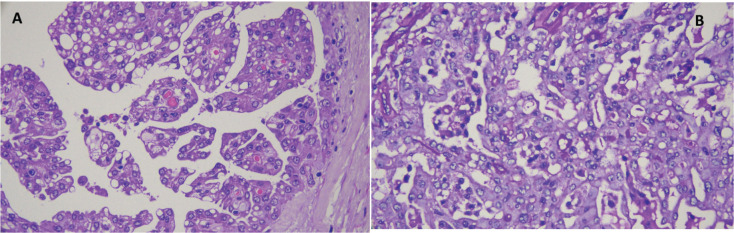
(a): Haematoxylin-Eosin staining (10×) shows epithelial cells arranged in a papillary pattern with vacuoles in the cytoplasm that determine the formation of cystic units with eosinophilic interior content. (b): Epithelial cells with PAS-positive inclusions in the cytoplasm (40×).

**Figure 7.**
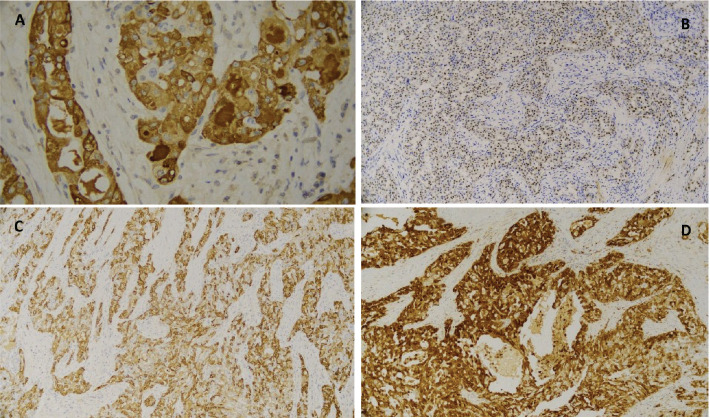
Immunohistochemical findings typical of MASC. (a): Tumour cells showed positive expression for the nuclear and membrane proteins mammaglobin, (b): GATA and (d): S100. (c): Phenotypic expression of mammaglobin, GATA 3 and S100 was also observed.

Microscopic study of the surgical specimen identified a lesion measuring 27 × 22 mm. It was characterised by proliferation of neoplastic cells, with moderate atypia, macrokaryosis, occasional nucleoli and abundant cytoplasm with a vacuolated appearance arranged in a predominantly microcystic and follicular pattern, and with fine fibroconnective septa (Figure 6). There was no perineural or lymphovascular infiltration nor extraparenchymal extension. Surgical margins were free of tumour lesions. Neoplastic cells were positive for S100, mammaglobin, periodic acid Schiff (PAS) and GATA 3 (Figure 7a–d). Fluorescence in situ hybridisation (FISH) analysis was performed on the neoplastic cells and rearrangement of the ETV6 gene was observed. Genetic, immunohistochemical, morphological characteristics were consistent with MASC.

Postoperative adjuvant therapy was not considered necessary after surgical resection. The patient had regular clinical follow-ups (monthly for 1 year, then every 2 months), local and regional ultrasound every 6 months and annual CT scans with intravenous contrast. The patient is currently free of disease (disease-free period: 36 months) and has experienced no sequelae from surgery.

## Discussion

MASC is a recently described tumour of the salivary glands, with a distinctive, characteristic t(12;15) (p13;q25) ETV6-NTK3 chromosomal translocation, which leads to fusion of the ETV6 gene on chromosome 12 and the NTRK3 gene on chromosome 15 [7]. ETV6-NTRK3 gene fusion codes for a chimeric tyrosine kinase that shows altered activity in salivary gland epithelial and myoepithelial cells [7].

Since the work of Skálova et al [3], MASC has been recognised as a new diagnostic entity in a wide range of head and neck tumours. Many case reports and retrospective studies have appeared since that publication, identifying approximately 660 published cases of MASC [8]. Most of these cases report an average age at onset of 40–50 years, but with a range that includes children, adolescents and older adults. MASC is slightly more common in males, but there are no significant differences between the sexes and there is no racial predisposition.

MASC mainly affects the parotid gland but can affect the submaxillary and minor salivary glands, mainly in the oral cavity. Lesion size varies, lesion growth is slow and insidious, and the most common presentation is a palpable tumour. MASC generally appears as a low-grade, malignant lesion with a low rate of regional lymph node metastases and a low mortality rate. [9].

The most common differential diagnoses of these tumours are acinic cell carcinoma, mucoepidermoid carcinoma and adenocarcinoma [5, 10]. MASC has often been diagnosed as acinic cell carcinoma [2, 3]. However, the immunohistochemical and molecular profile of MASC makes it possible to distinguish it from other salivary gland tumours and diagnose it. MASC shows a diffuse and strong expression of pancytokeratins (AE1-AE3 and CAM 5.2), CK7, CK8, CK18, CK19, epithelial membrane antigen, S-100 protein and vimentin. Tumour cells always show strong, positive expression of signal transducer and activator of transcription 5a and mammaglobin. MASC is also often positive for Gross cystic disease fluid protein 15 (GCDFP-15). Basal and myoepithelial cell markers such as p63, calponin, CK14, actin and CK5/6 are nearly always negative [2]. This immunohistochemical profile makes it possible to establish the suspected MASC diagnosis, then confirm it by using FISH to verify ETV6-NTK3 gene fusion.

Like secretory mammary carcinoma, MASC has a recurrent chromosomal translocation, t(12;15) (p13;q25), that results in the fusion of the ETV6 gene on chromosome 12 and the NTRK3 gene on chromosome 15. The biological consequence of this translocation is the fusion of the transcriptional regulator ETV6 with the membrane receptor NTRK3. Fusion triggers activation of this kinase, which promotes cell proliferation. ETV6-NTRK3 gene fusion has not been shown in any other salivary gland neoplasm [2, 3].

Management of MASC now follows the traditional guidelines used in treating malignant salivary gland neoplasms, the basis of which is oncological surgical resection. Lymphadenectomies, radiation and chemotherapy have been reserved for cases with regional involvement, histological risk factors (positive margins, perineural invasion) or metastatic disease [4]. However, there are no treatment guidelines for MASC due to its rarity, low numbers of cases and lack of randomised studies to provide clear evidence. Most evidence comes from case reports in which the main treatment was primary superficial and/or deep parotidectomy and lymphadenectomy in the subset of patients with high-grade transformation or regional involvement.

Most MASC cases involve low-grade malignant neoplasms with effective surgical management, but there are case reports of high-grade, more aggressive cases with higher rates of recurrence [11]. Some MASCs with low-grade morphology may behave more aggressively and require other treatment in addition to surgery [12]. Recent research by Skalova *et al* [3] identified several molecular alterations that treatments could target, specifically, a mutation in the VIM-RET gene. The VIM gene codes for vimentin, an intermediate filament protein that forms part of the cellular cytoskeleton. It has been suggested that overexpression of vimentin indicates the onset of epithelial–mesenchymal transformation during progression to more aggressive tumours. The RET gene codes for a membrane receptor tyrosine kinase protein. VIM-RET fusion preserves the tyrosine kinase domain, which could, in certain cases, be an objective of treatments targeting RET [13].

More research is required to gather accurate data on the clinical behaviour and long-term prognosis of MASC. Standardised guidelines are also required for surgical treatment, the role of radiotherapy and chemotherapy, the time and manner of follow-up, regional lymph node involvement, distant metastases, recurrence and mortality associated with MASC [5, 10].

## Conclusion

MASC is a recently described and rare salivary gland tumour. Few cases have been reported worldwide and there are no definitive studies that make it possible to establish the prognosis and behaviour of MASC. Resection with adequate oncological margins is the basis of treatment, but more information is needed to establish evidence-based treatment guidelines.

## Informed consent

The patient’s prior informed consent was obtained for this case report.

## Conflicts of interest

There are no conflicts of interest to declare in this publication.

## Funding

The listed authors did not receive funding for preparing this manuscript.
